# Rapid measurement of hydrogen sulphide in human blood plasma using a microfluidic method

**DOI:** 10.1038/s41598-019-39389-7

**Published:** 2019-03-01

**Authors:** R. Karunya, K. S. Jayaprakash, R. Gaikwad, P. Sajeesh, K. Ramshad, K. M. Muraleedharan, M. Dixit, P. R. Thangaraj, A. K. Sen

**Affiliations:** 1 Department of Mechanical Engineering, Indian Institute of Technology Madras, Chennai, 600036 India; 2 Department of Chemistry, Indian Institute of Technology Madras, Chennai, 600036 India; 3 Department of Biotechnology, Indian Institute of Technology Madras, Chennai, 600036 India; 4 Department of Cardiothoracic Surgery, Apollo Hospital, Chennai, 600006 India

## Abstract

Hydrogen sulfide (H_2_S) is emerging as an important gasotransmitter in both physiological and pathological states. Rapid measurement of H_2_S remains a challenge. We report a microfluidic method for rapid measurement of sulphide in blood plasma using Dansyl-Azide, a fluorescence (FL) based probe. We have measured known quantities of externally added (exogenous) H_2_S to both buffer and human blood plasma. Surprisingly, a decrease in FL intensity with increase in exogenous sulphide concentration in plasma was observed which is attributed to the interaction between the proteins and sulphide present in plasma underpinning our observation. The effects of mixing and incubation time, pH, and dilution of plasma on the FL intensity is studied which revealed that the FL assay required a mixing time of 2 min, incubation time of 5 min, a pH of 7.1 and performing the test within 10 min of sampling; these together constitute the optimal parameters at room temperature. A linear correlation (with *R*^2^ ≥ 0.95) and an excellent match was obtained when a comparison was done between the proposed microfluidic and conventional spectrofluorometric methods for known concentrations of H_2_S (range 0–100 µM). We have measured the baseline level of endogenous H_2_S in healthy volunteers which was found to lie in the range of 70 μM – 125 μM. The proposed microfluidic device with DNS-Az probe enables rapid and accurate estimation of a key gasotransmitter H_2_S in plasma in conditions closely mimicking real time clinical setting. The availability of this device as at the point of care, will help in understanding the role of H_2_S in health and disease.

## Introduction

Gasotransmitters, nitric oxide (NO), carbon monoxide (CO), and hydrogen sulfide (H_2_S) are emerging as potentially key signalling molecules and may have important roles in physiology^1–3^. Endogenous hydrogen sulfide appears to have an important role in maintaining endothelial homeostasis which in turn contributes to normal functioning of a variety of organs^1–5^. Hydrogen sulfide may act as both an anti-inflammatory and pro-inflammatory agent in animal models and cell culture^3,6^. It also acts as cytoprotective agent against oxidative stress in nervous and cardiovascular systems^7^.

Hydrogen sulfide is soluble in water, and hence in plasma, and exists in plasma and extracellular matrix as approximately, 20% H_2_S, 80% HS^−^ ion and a very negligible amount as S^2−^ at a pH of 7.4^5^. The percentage composition of sulphide in plasma is sensitive to temperature and pH. All the three forms of dissolved hydrogen sulfide can be commonly termed as sulfide. This sulfide can exist in free form, which is commonly termed as free sulfide, as well as in bounded form as sulfates, sulfide, sulfonates and elementary sulphur^5^. There are various methods available to detect the amount of free sulfide and bound sulfide. Bound sulfide can be measured only when it is released from its bounded form, i.e. in acidic environments^5^. H_2_S level in plasma is influenced by its interaction with blood constituents such as RBC and plasma proteins^4,8–10^.

Colorimetric methods, such as methylene blue test^11^, absorbance based techniques^12–14^ and chromatography^15,16^ based detection have been the accepted methods for the measurement of both the forms of sulfide. There are two different methods of measurement in methylene blue test: direct and indirect method. Presence of reducing agents in samples may interfere with the test and colour formation, which limits its application in plasma^5^. Electrochemical methods using ion-selective electrodes have also been developed to quantify sulfide in aqueous environments^17,18^. The main drawback of the method is that, in most of the techniques, the ion-selective electrodes are specific to the S^2−^ ions, but the percentage proportion of S^2−^ ions in physiological conditions (~7.4 pH) is negligible and requires a highly alkaline environment (pH > 12.0) for its proportion to reach detectable limits^19^. There have been attempts on direct measurement of the dissolved H_2_S using electrochemical techniques^20,21^. In one of the techniques, dissolved H_2_S present in an aqueous medium is permeated through a silicone membrane to reach the working electrode (where current is generated) immersed in an electrolyte solution^20^. This method detects only the H_2_S in the solution and not the HS^−^ ions which forms the 80% composition in the pH range 7.0 to 8.2 and solely depends on the membrane permeability and the sensor dimensions^20^. Very recently, direct measurement of H_2_S at physiological pH using electrochemical method was investigated^22^. The problem associated with the method is that the oxidation of H_2_S results in elemental sulphur which has to be removed from the surface of the electrode for repeated measurements^22^. Fluorimetric detection of sulfide have gained attention recently and new fluorophores have been developed that selectively react with sulfide, despite the presence of other species, to form a fluorescent compound^3,23^. Different fluorophores have been developed to detect and measure the amount of sulfide in bulk solutions as well as in living cells. A review by Lin *et al*.^23^ summarises the different fluorescent probes for measurement of sulfide. Measurement of sulfide in fluids^14,24–26^ and living cells^13,27–29^ has been usually performed using bulk sample making it unwieldy for clinical use. However, by miniaturizing the detection and measurement system, it would be possible to usher in point of care devices for gasotransmitters. This would help in a more accurate estimation of these short lived volatile substances. In addition, miniaturisation would offer other potential advantages such as reduced reagent and sample consumption, smaller footprint and easier integration of electronic components. Considering the above, colorimetry and flourimetry are the two potential techniques that would satisfy the requirements.

There have been attempts to measure and monitor hydrogen sulfide in biosamples such as, microdialysis effluents^30^ and artificial cerebrospinal fluids^31^, albeit without miniaturisation. This was done by nanoparticle conjugation with a fluorophore, incubated with the sample in droplets in a PTFE tube and detected off chip by a commercially available real-time PCR instrument. To the best of our knowledge, there has been no attempt to develop a miniaturized method for detection of hydrogen sulphide in blood plasma.

## Device Design and Description

**Figure 1:**
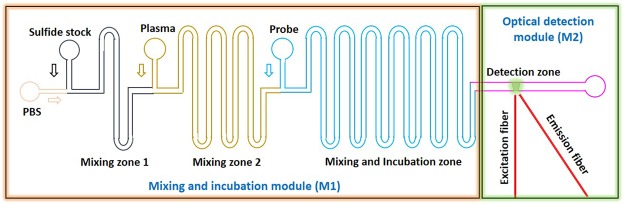
A schematic of the proposed microfluidic device, the mixing and incubation module and the detection module are shown.

A schematic of the proposed microfluidic device is shown in Fig. 1. The device comprises of two modules – mixing and incubation module, M1 and optical detection module, M2. In the mixing and incubation module M1, plasma (containing the sulfide) and chemical probe are mixed and incubated for a duration of 8 min (i.e. the time required for homogeneous mixing and incubation). The chemical probe, dansyl azide (DNS-Az), on reaction with sulfide forms dansyl amine, which is a fluorescent compound that is detected.

The mixing zone is designed to ensure complete mixing and incubation of the sample and probe within the available channel length by taking into consideration the diffusion coefficients, the flow rates of the sample and probe and the cross section of the channel (see simulation data in the Supporting Information). To obtain different exogenous sulphide samples in phosphate buffered saline (PBS), first, the 100 μM stock solution of sulfide is mixed with (PBS) on-chip in the first mixing zone (Fig. 1). The diffusion coefficient of sulfide solution in water is taken as D_s_ = 1.41 × 10^−5^cm^2^/s. The dimension of the channel is fixed as 200 μm × 200 μm. So the diffusion time (τ) is calculated as, ${\rm{\tau }}=({{\rm{w}}}^{2}/{{\rm{D}}}_{{\rm{s}}}) \sim 30\,{\rm{s}}$, where w is the width of the channel, D_s_ is the coefficient of diffusion of sulfide with water. The net flow rate of the sulfide stock solution and PBS mixture is fixed to at 22.5 μL/min, in which the flow rate of sulfide stock solution and PBS are controlled individually to obtain different concentrations of sulfide (see Table 1 in methods section). Hence the velocity (u) of sulfide and PBS mixture is 9.38 × 10^−3^ m/s, which gives a mixing length L = τu of 0.30 m. Next, the probe is infused at a rate of 2.5 μL/min, resulting in a total flow rate (sulfide sample and probe) of 25 μL/min, is mixed with the sulfide sample in the mixing and incubation zone. The diffusion coefficient of ethanol (which is the solvent used for the probe DNS-Az) in water is taken as 1.23 × 10^−5^ cm^2^/s, which gives rise to a diffusion time of 35 s and a mixing length of 0.35 m. Then the FL assay in buffer (sulfide sample + probe) is incubated on-chip for 5 min and detected in the detection module. The length of the mixing and incubation zone is such that it takes care of the mixing time of 35 s and the incubation time of 5 min.

**Table 1:** Different combinations of flow rates of various samples used for sulfide detection in PBS, probe: DNS-Az, Sulfide stock solution of 100 μM.

Exogenous concentration of sulfide (µM)	PBS flow rate (µL/min)	Sulfide stock flow rate (µL/min)	Probe flow rate (µL/min)
Baseline	22.500	0	2.50
15	19.125	3.375	2.50
25	16.875	5.625	2.50
50	11.250	11.25	2.50
75	5.625	16.875	2.50
100	0	22.500	2.50

To obtain different exogenous sulfide samples in plasma, first, the 145 μM stock solution of sulfide is mixed with PBS (buffer) on-chip in the first mixing zone for 30 s. The individual flow rates of sulfide stock and the PBS is varied to obtain different concentrations of sulfide, which is detailed in Table 2 in methods section. The net flow rate of this mixture is maintained at 15.75 μL/min. Subsequently, for mixing of the PBS containing sulphide and undiluted plasma at a rate of 6.75 μL/min (which contains 90–95% water), additional mixing time of 30 s. and mixing length of 0.35 m will be required which is shown in Fig. 1 as mixing zone 2. Finally, the exogenous sulphide spiked plasma is mixed and incubated with the fluorescent probe for 35 s and 5 min respectively.

**Table 2:** Different combinations of flow rates of various samples used for sulfide detection in plasma, probe: DNS-Az, Sulfide stock solution of 145 μM.

Exogenous concentration of sulfide (µM)	PBS flow rate (µL/min)	Sulfide stock flow rate (µL/min)	Undiluted plasma flow rate (µL/min)	Probe flow rate (µL/min)
Baseline	22.50	0	0	2.50
0	15.75	0	6.75	2.50
15	12.88	2.87	6.75	2.50
25	11.13	5.62	6.75	2.50
50	7.87	7.87	6.75	2.50
75	3.93	11.81	6.75	2.50
100	0	15.75	6.75	2.50

After the mixing and incubation module, the sample-probe mixture (fluorescent compound) is introduced into the optical detection zone. Here, the sample inside the channel is excited with a UV laser, 20 mW power, at 360 nm (PSU-H-FDA, Changchun New Industries Optoelectronics Tech. Co. Ltd., China) wavelength, using an excitation fibre. Upon excitation with UV, the fluorescent compound emits light in the wave length range 450 nm to 600 nm with the emission peak at 510 nm^25^. Multimode optical fibres (62.5/125 μm) were placed in the fibre grooves of the device adjacent to the channel. The excitation fibre is placed perpendicular to the flow direction and emission fibre is placed at 45° to the excitation fibre. The fluorescence intensity from the emission fibre is read in terms of number of photon using a Single Photon Counting Module (SPCM) from Thorlabs Inc., USA. To cut-off the excitation source, long pass optical filter of 410 nm (Thorlabs Inc., USA) was used in the collection fibre just before the signal arrives the SPCM.

## Experimental

Experiments were performed to measure sulfide in two different samples namely, buffer (PBS) and plasma, using three different methods of detection. The first method makes use of the conventional fluorescence measurement using a standard spectrofluorometer, the second method includes the preparation of the FL assay and detection using the microfluidic detection module M2 and the third method comprises the preparation of the assay using the mixing and incubation module M1 and detection using the detection module M2. First, known concentrations of sulfide, in the range 0 μM to 100 μM, was mixed with probe and incubated in buffer and measured using the spectrofluorometer (conventional method). The same concentrations of sulfide in buffer were measured by externally mixing the buffer with sulfide and probe (off-chip mixing) and detected using the microfluidic detection module (on-chip detection). Next, the buffer, sulfide stock solution and the probe are mixed and detected using the proposed device (on-chip mixing and detection). Similarly, known concentrations of sulfide is added (exogenous sulfide) to plasma and the detection is carried out using the different methods. Finally, the proposed device was used to measure the levels of sulfide present in the plasma of healthy volunteers (i.e. measure endogenous sulfide levels in blood plasma).

To carry out measurements using the device, the sample, the probe and the sulfide stock solution were taken in separate 1 mL syringes and infused into the microchannel device using a syringe pump (Cetoni GmbH, Germany) at specified flow rates as mentioned in Tables 1 and 2. The different combinations of flow rates of sample and reagents used for the detection of sulfide in PBS and plasma (after three-fold dilution) are shown in Tables 1 and 2 respectively. The flow rate of the mixture was maintained at 25 μL/min in all cases. The sample, sulfide stock and probe flow rates were varied to maintain the appropriate concentrations, in the range 0 μM to 100 μM.

## Results and Discussions

### Detection of sulfide in PBS

Different volumes of PBS and sulphide (from the stock of 100 µM sulfide) were mixed to obtain a total sample volume of 900 μL with sulphide concentration varying in the range 0 to 100 µM. Then, 100 μL of DNS-Az was added to this sample and mixed to get a final probe concentration of 200 µM in a net volume of 1.0 mL. To determine the effect of mixing and incubation time on the fluorescence intensity, two different samples of sulfide concentration 100 µM in 1.0 mL of PBS were taken. In one of the samples, the probe and sulphide thoroughly mixed externally using a vortex mixer for ~30 s while in the other sample, the probe was just added to the sulphide but was not mixed. For both the samples, the fluorescence intensity was measured using a spectrofluorometer at different time points after the probe was added to the sulphide. The sample was subjected to an excitation at 360 nm with a slit width of 5 nm and the emission was recorded over a range from 400 nm to 650 nm with a slit width of 5 nm.

**Figure 2:**
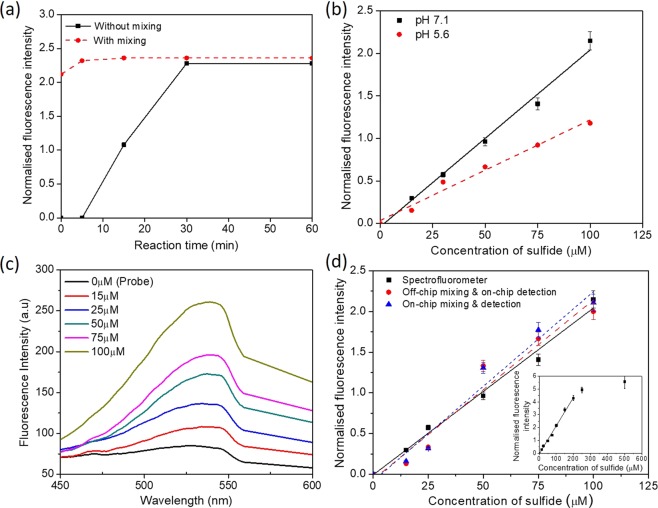
(**a**) Effect of mixing and incubation time on the FL intensity, sulfide concentration 100 µM in 1.0 mL of PBS, in one of the samples, probe and sulphide thoroughly mixed externally using a vortex mixer for ~30 s and in the other the probe just added to sulphide but not mixed, (**b**) Effect of pH on the change in fluorescence intensity at different sulphide concentrations one with a pH of 7.1 and other with a pH of 5.6, (**c**) Variation of fluorescence spectrum with concentration of sulphide in PBS, (**d**) Effect of sulphide concentration in PBS on the FL intensity measured using spectrometer, off-chip mixing and on-chip detection, and on-chip mixing and detection, probe concentration 200 μM in all cases, the inset shows the change in fluorescence intensity for the range of 0 µM to 500 µM of sulfide measured in spectrofluorometer. For each data point, the error bars represent the standard deviation from three different readings.

Figure 2a shows the normalized fluorescence intensity at different time points. The normalised intensity is calculated as (*I* − *I*_0_)/*I*, where *I* is the fluorescence intensity for a given sulfide concentration and *I*_0_ is the baseline intensity of the probe. The probe, DNS-Az, has a weak emission band with λ_max_ at 530 nm whereas dansyl amine is more emissive with λ_max_ varying between 530 nm to 540 nm (depending on the sulphide concentration). The fluorescence intensity was read as photon counts using the single photon counting module. For each reading, the signal was recorded hundred-times with an integration time of 100 ms and the average was taken as the intensity *I*. For the case in which the probe was just added to the sulphide, since mixing is purely due to molecular diffusion, the normalized intensity increases with time and attains a steady value at *t* = 30 min. For the case in which the sulphide and probe were thoroughly mixed, there was only a small increase in the intensity observed till *t* = 5 min beyond which a steady intensity value was obtained (Fig. 2a). This indicates that, after thorough mixing of the sulphide and probe, the required incubation (reaction) time is 5 min.

Next, the effect of pH on the change in the fluorescence intensity at different sulphide concentrations was studied with two samples of different pH values, pH 5.6 and 7.1, as shown in Fig. 2b. In both the cases, a linear increase in the fluorescence intensity with time was observed (with *R*^2^ ≥ 0.95). As seen, the intensity is higher for the sample of pH 7.1 as compared to that of pH 5.6, which can be attributed to the fact that in acidic conditions (pH 5.6), the amount of HS^−^ ions decreases^5,32,33^. In samples of higher pH, more free H_2_S gets dissolved and thus the percentage of HS^−^ ions increases until pH = 8.2 beyond which the HS^−^ ions saturate^5,32,33^. So, for the rest of the study, the pH of the sample is maintained at 7.1. Also, the pH 7.1 is closer to the physiological pH of 7.4, which is relevant for the testing of real-time clinical plasma samples.

Further, variation of fluorescence spectrum with the concentration of sulphide in PBS was studied using the spectrometer and the results are shown in Fig. 2c. For each measurement, 1.0 mL of PBS and sulphide mixture was taken and mixed with DNS-Az so the concentration of probe in the mixed sample is 200 μM. The solution was mixed thoroughly off-chip using a vortex mixer for 30 s and incubated for 5 min in a micro-centrifuge tube. The mixed sample was then taken in a cuvette and fluorescence intensity was measured using a spectrometer. The sulphide concentration was varied as 0 μM (which gives the baseline fluorescence intensity of the probe), 15 μM, 25 μM, 50 μM, 75 μM and 100 μM. The peak fluorescence intensity was observed between 530 nm to 540 nm. For each sulphide concentration, the peak intensity values are taken and plotted in Fig. 2d (square symbols), which shows a linear increase in fluorescence intensity with the sulphide concentration. The inset in Fig. 2d shows a linear response of FL intensity, measured using the spectrofluorometer, for the sulphide concentration in the range 0 µM to 200 µM, beyond which the response becomes non-linear. The nonlinearity beyond 200 µM can be attributed to the fact that the stoichiometric ratio of the probe with sulfide is 1:1 and since the probe concentration is fixed at 200 µM, addition of sulfide more than 200 µM did not result in appropriate increase in the intensity.

To demonstrate on-chip detection, PBS samples at the same sulphide concentrations (in the range 0 to 100 μM) were taken and mixed and incubated with the probe outside the device (off-chip) and fluorescence intensity was measured using the detection module of the device. The channel cross section, flow rate, diameter of the fibre core and the distance between the fibre and the channel can affect the number of amine molecules present for excitation over a small duration of time. There should be adequate number of molecules present over a finite time to provide detectable florescence signal. Microchannels of different cross-sections were used and it was found that channel cross-section of 300 μm × 200 μm (width × height) offers better response (signal) compared with channels of smaller dimensions thus selected for the study. For example, in case of channels of smaller cross-section, say 100 μm × 100 μm, at flow rates of 10 µL/min to 25 µL/min and sulfide concentration of 15 µM, the FL intensity was inadequate for detection. The beam width of the excitation is decided by the fiber core diameter and distance between the channel and fiber. Increasing this distance would increase the beam width but decrease the intensity of the excitation incidenting on the fluorophore molecules. The distance between the emission fiber and the channel would have a similar effect. In experiments, the distance between the fiber (excitation and emission) and the channel was kept fixed at 50 µm and the flow velocity was maintained at 6.94 mm/s. The beam width of the excitation is ~200 µm and thus the average time spent by the molecules in the detection zone is ~30 ms. This shows that for dansyl amine, 30 ms is sufficient to obtained a considerable change in the FL signal. The net flow rate was fixed at 25 μL/min. Our experiments show that flow rate, in the range of 10 μL/min to 50 μL/min, does not have any considerable effect on the FL intensity. Figure 2d (circle symbols) shows the normalised fluorescence intensity for different concentrations of sulfide in PBS measured using the detection module.

To demonstrate on-chip mixing and incubation and detection, the sulfide stock, PBS and probe are infused through the three separate inlets into the mixing and incubation module. As discussed in section 2, the sulphide stock and PBS are mixed in a serpentine channel (mixing zone 1 and 2 in Fig. 1) for a duration of 30 s to ensure thorough mixing. Then, the sulphide + PBS sample was mixed with the fluorescent probe in the second serpentine channel in the mixing and incubation zone for a duration of 6 min to ensure thorough mixing and for the reaction to complete. Table 1 summarises the flow rates of sample and reagents to obtain different concentrations of sulfide, in the range 0 μM to 100 μM. Figure 2d shows the variation in fluorescence intensity with the sulphide concentration measured in the integrated microfluidics device. As observed, the characteristics obtained using the three different approaches are linear (with *R*^2^ ≥ 0.95) and match well in terms of the normalised FL intensity, with a standard error of 0.121, which validates the design of the mixing and incubation and detection modules of the microfluidic device.

### Detection of sulfide in plasma

To measure the exogenous sulphide in plasma, first, different volumes of sulphide stock solution (145 μM) and PBS are taken and mixed to get a sulfide solution. For each measurement, the centrifuged plasma and the sulfide solution were mixed (at different ratios, depending on the required concentration of sulfide) to a final sample volume of 900 μL and then 100 µL of DNZ-Az probe was mixed with the sample to obtain a final probe concentration of 200 µM. The sulphide and probe mixture sample was excited at 360 nm with a slit width of 5 nm and the emission was recorded between 400 nm to 650 nm with a slit width of 2.5 nm. In plasma, the peak fluorescence intensity was observed at 490 nm.

**Figure 3:**
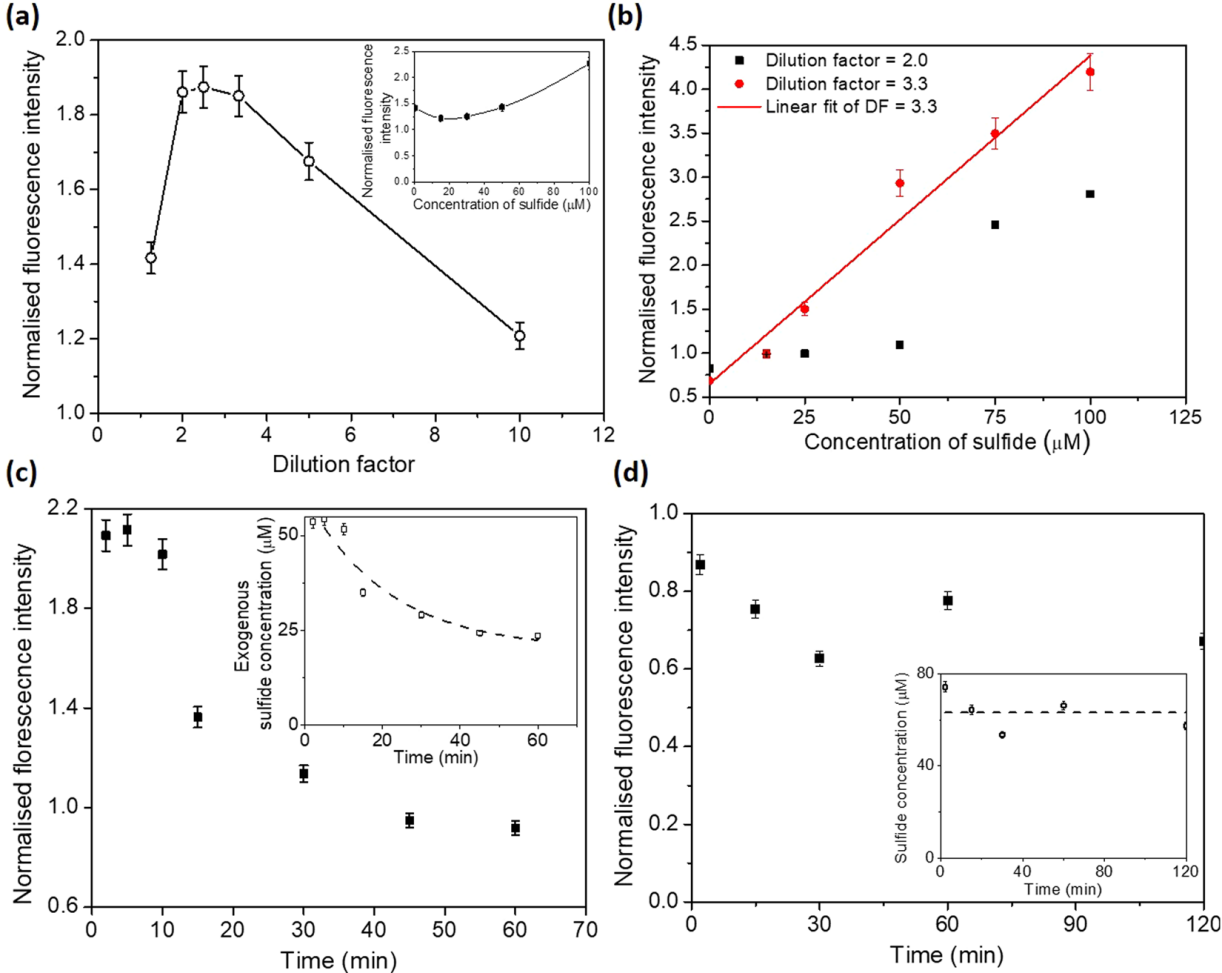
(**a**) Effect of dilution of plasma (containing endogenous sulphide) without the addition of exogenous sulphide in plasma on the fluorescence intensity, probe concentration 200 µM, inset shows the change in FL intensity with addition of sulfide in undiluted plasma, (**b**) Variation of FL intensity with exogenous sulphide in diluted plasma of different dilution factors (2.0 and 3.3), (**c**) Decay of FL intensity measured at different time points after exogenous sulphide added to three-fold diluted plasma, inset shows the corresponding sulphide concentration with time, (**d**) Change in the FL intensity of the endogenous sulfide in three-fold diluted plasma with time, probes added to diluted plasma at different time points, mixed thoroughly and detected immediately, probe concentration 200 µM in all cases. For each data point, the error bars represent the standard deviation from three different readings.

First, the endogenous sulfide inherently present in plasma was measured and then exogenous sulfide was added to vary the concentration of sulphide in the plasma. Normally, when more sulphide is added to the plasma, the fluorescence intensity is expected to increase. To our surprise, we observed a decrease in FL intensity with increase in the sulphide concentration in undiluted plasma (see inset of Fig. 3a). Moreover, the decrease in FL intensity with increase in sulphide concentration (Fig. 2d) was not observed in case of PBS (non-plasma).

To reduce the interaction between human blood constituents and exogenously added sulphide, we performed experiments with plasma at different dilutions. Here, ‘dilution factor’ is defined as the ratio of the volume of the plasma + buffer to the volume of the plasma. Figure 3a shows the effect of dilution of plasma (containing endogenous sulphide), without the addition of exogenous sulphide in plasma, on the fluoresce intensity. The results show that fluorescence intensity increases up to a dilution factor of 3.3 and decreases with further increase in the dilution factor.

Diluted plasma (with endogenous sulphide level), at a dilution factor of 2.0, was spiked with exogenous sulphide in steps of 25 µM from 0 µM to 100 µM and the fluorescence intensity was measured using a spectrofluorometer. Figure 3b shows that at a dilution factor of 2.0, the relationship between fluorescence intensity and sulphide concentration is highly nonlinear. This indicates that, at a dilution factor of 2.0, the concentration of the proteins is such that there is a strong interference^4,8^ between the proteins and the exogenous sulphide that affect the measurements. However, when the dilution factor was increased to 3.3, a linear relationship (with *R*^2^ = 0.98) was observed as shown in Fig. 3b. The ability of plasma proteins to act as a sink^4,8^ for H_2_S appeared to be insignificant at a dilution factor of 3.3 from our experiments. With further increase in the dilution factor, there is a decrease in the sulphide concentration due to which the FL intensity decreases. Hence, we performed all subsequent studies at a dilution factor of 3.3.

We have performed experiments to study the time rate of change of consumption of exogenous sulphide in plasma. Diluted plasma at a dilution factor of 3.3 was taken in separate eppendorf tubes and each sample was spiked with sulfide of concentration 50 μM. After addition of sulphide, FL probe was added to each sample at different time points, varying from 2 min to 60 min and the FL intensity was measured using the spectrofluorometer and the results are presented in Fig. 3c. As observed, there is a small decrease in FL intensity (~3.5%) up to 10 min beyond which the FL intensity decays exponentially with time. The inset in the Fig. 3c shows the corresponding sulphide concentration at different time points (obtained using the calibration curve in Fig. 2d). The results also indicate that the concentration of the exogenous sulphide reduces to half of its initial concentration at 60 min. Similar study was carried out with PBS but in contrast to plasma, no significant change in the FL intensity was observed (within 60 min) due to the absence of plasma proteins (see Fig. 2a).

Next, after the sample collection and centrifugation, the FL probe was mixed with the plasma at different time points and FL intensity was measured using a spectrofluorometer. Figure 3d shows the change in the FL intensity for the endogenous sulfide (no spiked or exogenous sulphide) with time. It is observed that there is only a ~14% change in the FL intensity up to 120 min. The inset in Fig. 3d shows the corresponding sulphide concentration (obtained using calibration curve in Fig. 2d) at different time points. The average value of the measured sulphide concentration was found to be 71.85 µM with a standard deviation of 7.62 µM. So, we observe that the concentration of endogenous sulphide in plasma does not vary significantly with time whereas the concentration of exogenous sulphide added to the plasma gets scavenged by the proteins present in the plasma after 10 min.

**Figure 4:**
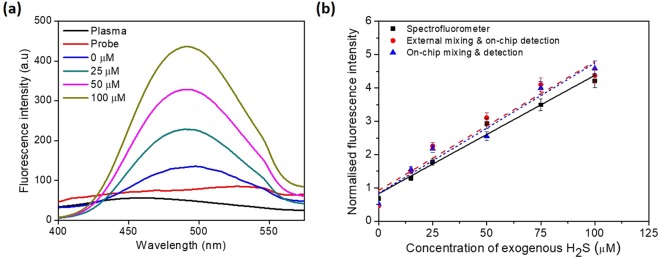
(**a**) Fluorescence spectrum obtained for different exogenous concentrations (0 to 100 µM) in plasma, (**b**) Linear response of the probe from three different schemes of detection in plasma, (**b**) Effect of exogenous sulphide concentration in plasma on the normalized FL intensity measured using spectrometer, off-chip mixing but on-chip detection, and on-chip mixing and detection, probe concentration 200 μM, sulphide and probe thoroughly mixed, pH = 7.1. For each data point, the error bars represent the standard deviation from three different readings.

Based on the outcome of the above studies, a microfluidic device was designed that facilitates complete mixing of the exogenous sulphide added to plasma with FL probe and detection within 10 min thus significantly reducing the influence of sulphide consumption by the plasma proteins. First, exogenous sulphide was added to plasma (from a healthy individual, see section 3) at a dilution factor 3.3, to obtain different sulphide concentrations and fluorescence signal was measured using a spectrofluorometer. Figure 4a shows the fluorescence spectrum obtained for different exogenous sulphide concentrations (0 to 100 µM) in plasma. Fluorescence spectra for plasma and probe obtained separately are also presented. The results show that the fluorescence signals obtained separately for the plasma and the probe (due to auto-fluorescence) over the spectrum are weak, as expected. The data corresponding to 0 µM is the endogenous sulfide present in plasma (but added with probe). The peak fluorescence intensity obtained using the spectrometer at different exogenous sulphide concentrations is presented in Fig. 4b (indicated by the square symbols).

Next, we proceed with experiments using the microfluidic device. First, to demonstrate the FL measurement using the detection module, exogenous sulfide at different concentrations (0 to 100 µM) was mixed with plasma (at a dilution factor 3.3) and incubated off-chip and detection was carried out on-chip. In the device shown in Fig. 1, the mixed sample was infused into the device through the PBS inlet and all other inlets were closed. Figure 4b shows the corresponding data showing the variation of FL intensity with exogenous sulphide concentration.

To demonstrate on-chip mixing and incubation and detection, sulfide stock of 100 μM, PBS and the undiluted plasma were taken separately and infused into the microfluidic chip (see Fig. 1). First, the sulfide stock and PBS were mixed inside the channel for 30 s in mixing zone 1 (see Fig. 1) and then the solution was mixed with the undiluted plasma for 60 s in mixing zone 2 such that the plasma in the resulting mixture is diluted by 3.3 fold with the sulfide solution. The flow rates of sulfide, PBS and plasma were adjusted (see Table 2 for details) to obtain the desired concentration of exogenous sulfide in plasma. The combined flow rate of the mixture of sulphide, PBS and plasma was maintained at 22.5 μL/min. The mixture was then mixed with probe (stock of 2000 μM) infused into the device at a flow rate of 2.5 μL/min, so that the final concentration of probe after mixing was 200 μM. The mixed sample was then incubated for 7.0 min in the device (mixing and incubation zone in Fig. 1) before it entered into the detection module at a flow rate of 25 μL/min.

**Figure 5:**
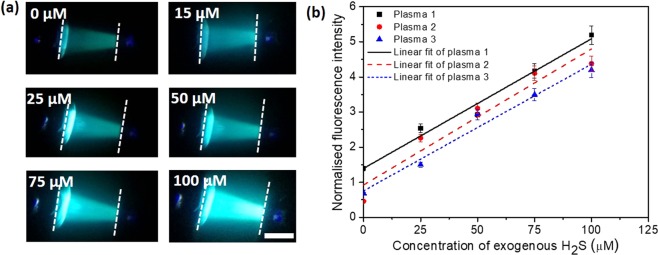
(**a**) Optical images of the fluorescence signal in the detection zone for different concentrations of exogenous sulfide (0 to 100 µM) in plasma obtained using a FL microscope, the scale bar represents 150 μm, (**b**) Variation of FL intensity with exogenous sulphide concentration for the plasma samples of different individuals, characteristic curves are linear (with *R*^2^ ≥ 0.92) in all cases with a slope of 0.0374 ± 0.0013 μM^−1^, inset shows concentration of sulfide measured from the linear fit to the exogenous sulphide added in the three plasma samples. For each data point, the error bars represent the standard deviation from three different readings.

Optical images of the fluorescence signals in the detection zone, for different concentrations of exogenous sulfide (0 to 100 µM), obtained using a FL microscope are presented in Fig. 5a. As observed, fluorescence signal is enhanced with increase in the sulphide concentration from 0 µM (endogenous sulphide level) to 100 µM. The FL intensity (peak value) obtained at different sulphide concentrations is presented in Fig. 4b. It is observed that the FL intensity versus sulphide concentration data obtained using three different approaches (i.e. from spectrofluorometer, off-chip mixing/incubation and on-chip mixing/incubation and on-chip detection) are linear (with *R*^2^ ≥ 0.93) and match well in terms of normalised FL with a standard error of 0.13. From the measured FL intensity (using the different approaches) at 0 µM sulphide, the endogenous sulphide concentration was found to be 76.28 ± 3.3 μM.

Next, blood samples from three healthy volunteers (2 males of age 26 and 27 years and 1 female of age 26 years) were collected (see section 3) and the corresponding plasma samples were separated (using centrifugation) and used for the study. The plasma samples were separately spiked with exogenous sulphide in the microfluidic device by adjusting the flow rates of sulphide stock + PBS and plasma (by following the protocol described above) to obtain exogenous sulphide concentration in the range 0 to 100 µM. Figure 5b shows the variation of FL intensity with exogenous sulphide concentration for the plasma samples of different individuals. The characteristic curves are found to be linear (with *R*^2^ ≥ 0.92) in all the three cases with a slope of 0.0374 ± 0.0013 μM^−1^. The intercept of the curves with the FL intensity axis provides the endogenous sulphide concentration, diluted by a factor of 3.3. By considering the dilution factor, the endogenous sulfide was determined to be in the range 70 μM to 125 μM. The slope of the curves is a measure of the change in normalised FL intensity for unit change in sulfide concentration (i.e. sensitivity) whereas the intercept provides the concentration of the endogenous sulfide present in the plasma of an individual which differs from person-to-person. The inset in Fig. 5b shows the measured sulfide concentration from the linear fit for the corresponding spiked exogenous sulfide. The sulphide concentration(combined HS^−^, H_2_S and S^2−^) in plasma measured using our device compares well with the values measured using different techniques reported in the literature, which falls in the range 50 to 100 μM^4,19,34,35^.

### Comparison between the conventional and proposed microfluidic methods

The real advantage of the proposed microfluidic method over the conventional method (using spectrometer) lies in the integration of the sample processing module for preparation of the assay and the detection module, thereby enabling automation. The proposed device can be coupled to a plasma separation module^36^ at the upstream of the mixing and incubation module to develop a microfluidic platform for continuous monitoring of hydrogen sulphide in plasma^37^. This would enable complete labour free process of assay preparation and measurement, in contrast to conventional method where a skilled personnel is required. Table 3 shows a comparison of both the methods in terms of the volume required for a single measurement. As observed, there is more than four-fold reduction in the volume required to perform the test. Reduction in the consumption of reagents directly reduces the cost per sample. The device foot-print can be reduced by mixing sample and probe in droplet microfluidics format that would eliminate the need of the long serpentine channel in the ‘mixing and incubation’ zone. The fluorescence signal can then be detected from the fluorophore present inside the droplets. Mixing and detection in droplet format would also require smaller volume of sample and reagent thus further reducing the volume requirement and cost. The costs of the instruments are comparable – the conventional technique would require a spectrofluorometer and a vortex mixer whereas the microfluidic set up would require a Laser source, SPCM, a filter and optical fibers. The cost of consumables such as centrifuge tubes, cuvettes, pipettes and pipette tips required for the conventional techniques would be similar to the cost of the chip. Further, unlike the conventional approach, in the proposed method, the assay is performed in a closed environment inside the microchannel device to enable more controlled measurements.

**Table 3:** Comparison of sample and reagent volumes required for the measurement of sulphide in plasma.

Sl. no.	Sample/ Reagents	Volume required in conventional method	Volume required in microfluidic method
1	Blood	1 mL	200 μL
2	Probe	100 μL	~25 μL
3	PBS	600 μL	~150 μL

## Conclusions

We reported a fluorescence (FL) based method for rapid measurement of hydrogen sulphide in plasma using a microfluidic method. The probe dansyl azide (DNS-Az) that is compatible with plasma and has a smaller reaction time when mixed with sulphide and on reaction sulfide forms dansyl amine, which is a fluorescent compound with excitation at 360 nm and emission peak at 530 nm. The effects of mixing and incubation time, pH, probe concentration and dilution of plasma on the FL intensity was studied which suggested a mixing time of 2 min followed by an incubation time of 5 min. The results showed a linear increase in FL intensity for sulphide in the range 0 to 200 μM beyond which the linearity was lost which could be attributed to the fact that the stoichiometric ratio of the probe with sulfide is 1:1 since the probe concentration was fixed at 200 µM. A decrease in FL intensity with increase in exogenous sulphide concentration in plasma was observed which could be due to the interaction between the proteins and sulphide present in plasma underpinning our observation. Experiments with diluted plasma showed that FL intensity initially increases up to a three-fold dilution but decreases thereafter. The plasma can buffer the exogenous sulphide rapidly below a critical dilution factor thus affecting the FL intensity. The results also reveal that detection of exogenous sulphide in diluted plasma (with three-fold dilution) needs to be performed within a duration of 10 min after which the sulphide concentration decreases exponentially with time indicating rapid buffering of sulphide. Sulphide present in buffer and plasma at different concentrations (in range 0 to 100 µM) was measured using the proposed microfluidic device and compared with that obtained from a spectrofluorometer, which showed excellent comparison and good linearity (with *R*^2^ ≥ 0.95). The proposed microfluidic device and method was also used for the measurement of endogenous sulfide and exogenous sulphide at different concentrations in plasma of different healthy individuals. With the proposed device, detection of diluted plasma has been performed over the concentration range 10 to 100 μM but since the plasma is diluted 3.3-times to reduce the plasma protein-sulphide interaction, the device can be used for detecting sulfide in plasma in the range 33 μM to 330 μM (i.e. the smallest and highest concentrations, respectively). The endogenous sulfide levels in plasma of healthy individuals were found to vary between individuals in the range of 70 μM to 125 μM, which agrees well with the values reported in literature. In summary, we have reported a microfluidic device with DNS-Az probe for rapid and accurate estimation of a key gasotransmitter H_2_S in plasma in conditions closely mimicking real time clinical setting. The availability of this device at the point of care, will help in understanding the role of H_2_S in health and disease.

## Materials and Methods

### Materials

Sodium sulfide (Na_2_S) flakes of molecular weight of 78 g/mol (AR grade) were purchased from Labochemie, Mumbai, India. Phosphate buffered saline (PBS) tablets of were purchased from Sigma-Aldrich, Bangalore, India and mixed with DI water ((Purelab, Elga LabWater, UK). Ethanol of 99% purity (HPLC grade), dansyl chloride of 99% purity (HPLC grade) and sodium azide of 99% purity (HPLC grade) were bought from Sigma-Aldrich, Bangalore, India. Dansyl chloride and sodium azide were used as the starting materials for DNZ-Az probe synthesis.

### Preparation of probe, stock solution and sample

Probe: DNS-Az probe (molecular weight of 276 g/mol) was synthesised and evaluated as reported by Peng *et al*.^25^ and stored at −5 °C. The probe was dissolved in 99% pure HPLC grade ethanol. The probe has a molecular weight of 276 g/mol. A probe stock solution of 2.0 mM concentration was prepared in ethanol that was mixed with sulfide solution at appropriate volumes during measurements such that the final concentration of the probe would be 200 μM irrespective of the concentration of sulfide to be measured.

### Sulfide Stock solution

Sodium sulphide flakes were used as the source of hydrogen sulphide. Two different stock solutions were freshly prepared, one with 100 µM of hydrogen sulfide and the other with 145 μM of hydrogen sulfide, by adding sodium sulphide flakes in 1X potassium buffer saline (PBS). The 100 μM sulfide solution was used as stock for detection of sulfide in buffer (PBS). Tween 80 of 2% w/w was added to the buffer for fluorescence enhancement. The 145 μM sulfide solution was used as stock for detection of exogenous sulfide in plasma.

### Plasma Preparation

Blood sample (1.0 mL) was collected in heparin coated vacutainers from healthy volunteers. Blood sample once collected was immediately centrifuged for 10 min at 6000 rpm. After centrifugation, the plasma (supernatant) was separated and collected. 1X PBS was used for dilution of plasma after centrifugation and collection.

### Sample preparation

For measurements in spectrofluorometer, a total sample volume of 1.0 ml was prepared and the fluorescence intensity was measured at different sulfide concentrations. In the 1.0 mL sample, the sulphide to the probe ratio was maintained as 9:1. Since our interest is to detect sulphide in the range from 0 μM to 100 μM, the maximum concentration of probe in the net volume was maintained as 200 μM. For the case of externally mixed and on-chip detection of sulfide, the same procedure was followed except that the detection was carried out on-chip (instead of the spectrometer).

### Device fabrication

Two different materials were used for fabricating the mixing and incubation and the detection modules. For mixing and incubation of the chemical species, silicon microchannel was used and detection was carried out in a poly methyl methacrylate (PMMA) microchannel. Silicon channels were used to avoid any chemical reaction of the probe (dissolved in ethanol) with the microchannels. Initially, we tried poly dimethyl siloxane (PDMS) and PMMA channels for the mixing and incubation but we observed swelling of PDMS and degradation of PMMA channels after long term exposure with the probe. Silicon wafers of 500 μm thickness was etched by deep reactive ion etching (DRIE) technique using standard protocols. Then, anodic bonding was used to seal the etched silicon channel layer with a planar glass. The silicone microchannel comprised serpentine channel configuration and has cross-section of 200 μm × 200 μm. The detection zone was machined in PMMA using a CNC micro-milling machine (Minitech Machinery, USA). A straight fluidic channel of cross section 300 μm × 200 μm was milled. Fibre grooves of cross-section 150 μm × 150 μm were machined to accommodate the excitation and collection fibres of outer diameter 125 μm. The fibre core which acts as the wave guide for the light is 62.5 μm. The milled PMMA channel was bonded with a planar PMMA substrate in a thermal hydraulic press operating at a temperature of 65 °C and pressure of 1.5 ton after exposing the surfaces to chloroform for a duration of 4 min. The mixing and the detection modules were connected using a polyurethane tubing of 580 µm ID.

### Author declaration

All methods were carried out in accordance with relevant guidelines and regulations. All experimental protocols were approved by Institute Ethics Committee, IIT Madras (Ref. No. IEC/2016/02/AK-1/01). Informed consent was obtained from all subjects or, if subjects are under 18, from a parent and/or legal guardian.

## Supplementary information

Supplementary Information
