# Supplementary material for: Rapid measurement of hydrogen sulphide in human blood plasma using a microfluidic method
Source: Sci Rep. 2019 Mar 1;9:3258. doi: 10.1038/s41598-019-39389-7 (PMC6397262; doi:10.1038/s41598-019-39389-7)
Supplement: Supplementary file 1 — Supplementary Information [file 41598_2019_39389_MOESM1_ESM.docx]

**Supplementary Information**

**Rapid measurement of hydrogen sulphide in human blood plasma using a microfluidic method**

R. Karunya^1^, K. S. Jayaprakash^1^, R. Gaikwad^1^, P. Sajeesh^1^, K. Ramshad^2^, K. M. Muraleedharan^2^, M. Dixit^3^, P. R. Thangaraj^4,1^ and A. K. Sen^1,*^

^1^Department of Mechanical Engineering, Indian Institute of Technology Madras, Chennai – 600036, India.

^2^Department of Chemistry, Indian Institute of Technology Madras, Chennai – 600036, India.

^3^Department of Biotechnology, Indian Institute of Technology Madras, Chennai – 600036, India.

^4^Department of Cardiothoracic Surgery, Apollo Hospital, Chennai – 600006, India.

^*^Author to whom correspondence should be addressed. Email: ashis@iitm.ac.in

**Design of the mixing zones**

The mixing zones in the microfluidic device design was verified using simulations (with Ansys Fluent). The following parameters were used for the simulations:

Channel: Serpentine rectangular channel of cross section $200 \mu m \times200 \mu m$ and length 0.24 m.

Mesh: Square mesh of size 10 μm.

Solver model: 3D, laminar, species transport.

Fluids: Water (sample) of viscosity and density equivalent to plasma and ethanol (solvent of the probe).

Diffusion coefficient: $D_{s}=1.41\times{10}^{-5} cm^{2}/s$. i.e. diffusion coefficient for mixing of ethanol (solvent for the probe) in water.

Boundary conditions: Water flow rate = 22.5 μL/min, ethanol flow rate = 2.5 μL/min.

Solution methods: Scheme – Simple

The Figure S1 below shows the channel (in Fig. S1a) and the simulation results (in Fig. S1b) in terms of the molar concentration of ethanol across the different cross-sections (along a line through the centre of the serpentine network). The serpentine channel has 12 turns, each of length 20 mm, making a total length of 0.24 m. The plot in the Fig. S1b represents the change in the molar concentration of ethanol across different sections. From the results, it is clear that at the 10^th^ turn of the channel, the solution becomes homogeneously mixed (indicated by the straight curve after the 10^th^ turn), which shows that a length of 0.2 m is sufficient for complete mixing of the liquids.


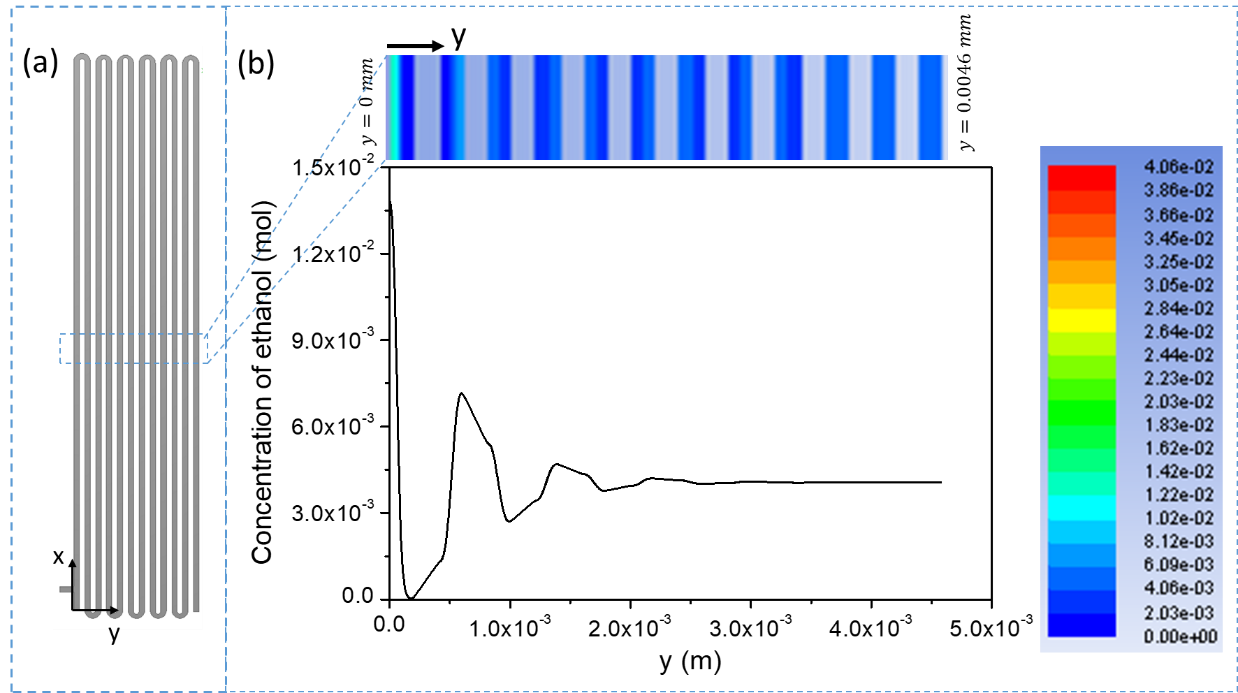


Figure S1: (a) Planar view of the serpentine channel taken for the simulations. (b) Plot shows the variation of the molar concentration of ethanol across different channel cross-sections along a line through the centre of the serpentine network. The corresponding contour plot is also shown.

Similarly, we have also simulated the mixing of sulfide stock solution in PBS (mixing zone 1 in the Figure 1 of the manuscript) and the concentration profile of sulfide across the different cross-sections shown below in Figure S2. The simulated length of the channel is 0.16 m (8 turns), whereas the actual length is 0.3 m, and the solution completely mixes with in the length of 0.14 m (7 turns) as shown in the contour profile in Figure S2.

Thus we assure that in the serpentine channel design, the sample, PBS and probe are completely mixed.


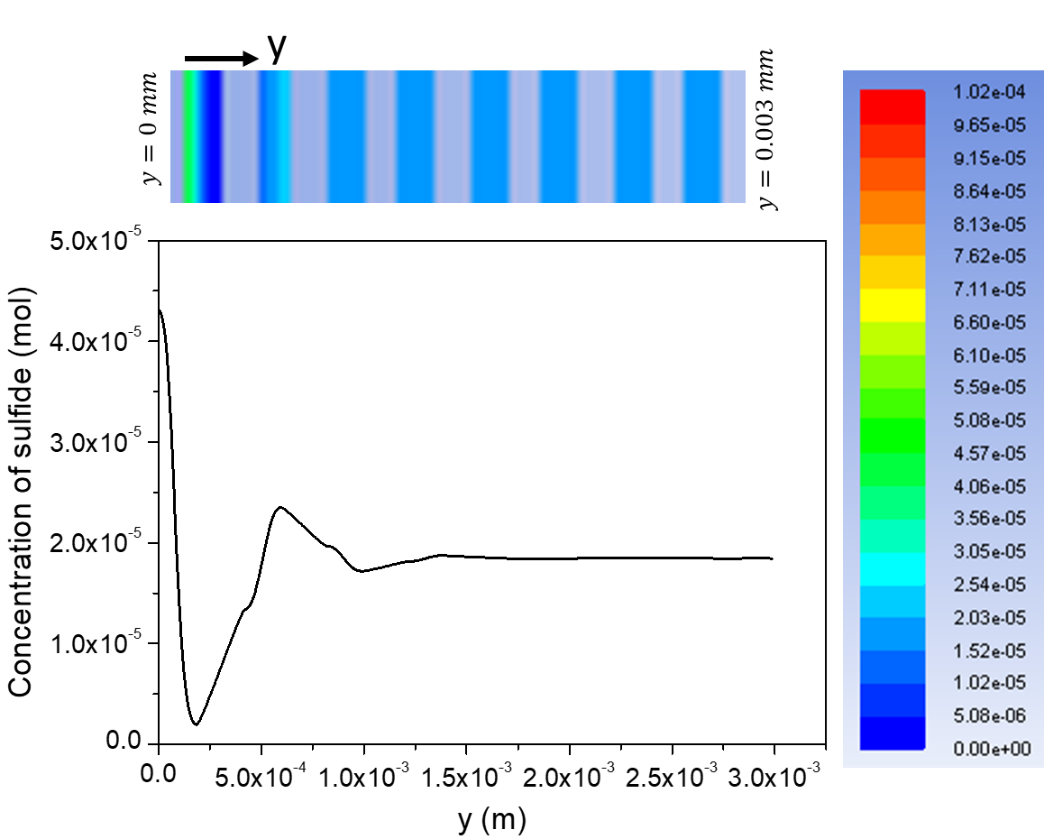


Figure S2: Plot shows the variation of the molar concentration of ethanol across different channel cross-sections along a line through the center of the serpentine network. The corresponding contour plot is also shown.
